# Supplementary figures and images for: Treemmer: a tool to reduce large phylogenetic datasets with minimal loss of diversity
Source: BMC Bioinformatics. 2018 May 2;19:164. doi: 10.1186/s12859-018-2164-8 (PMC5930393; doi:10.1186/s12859-018-2164-8)

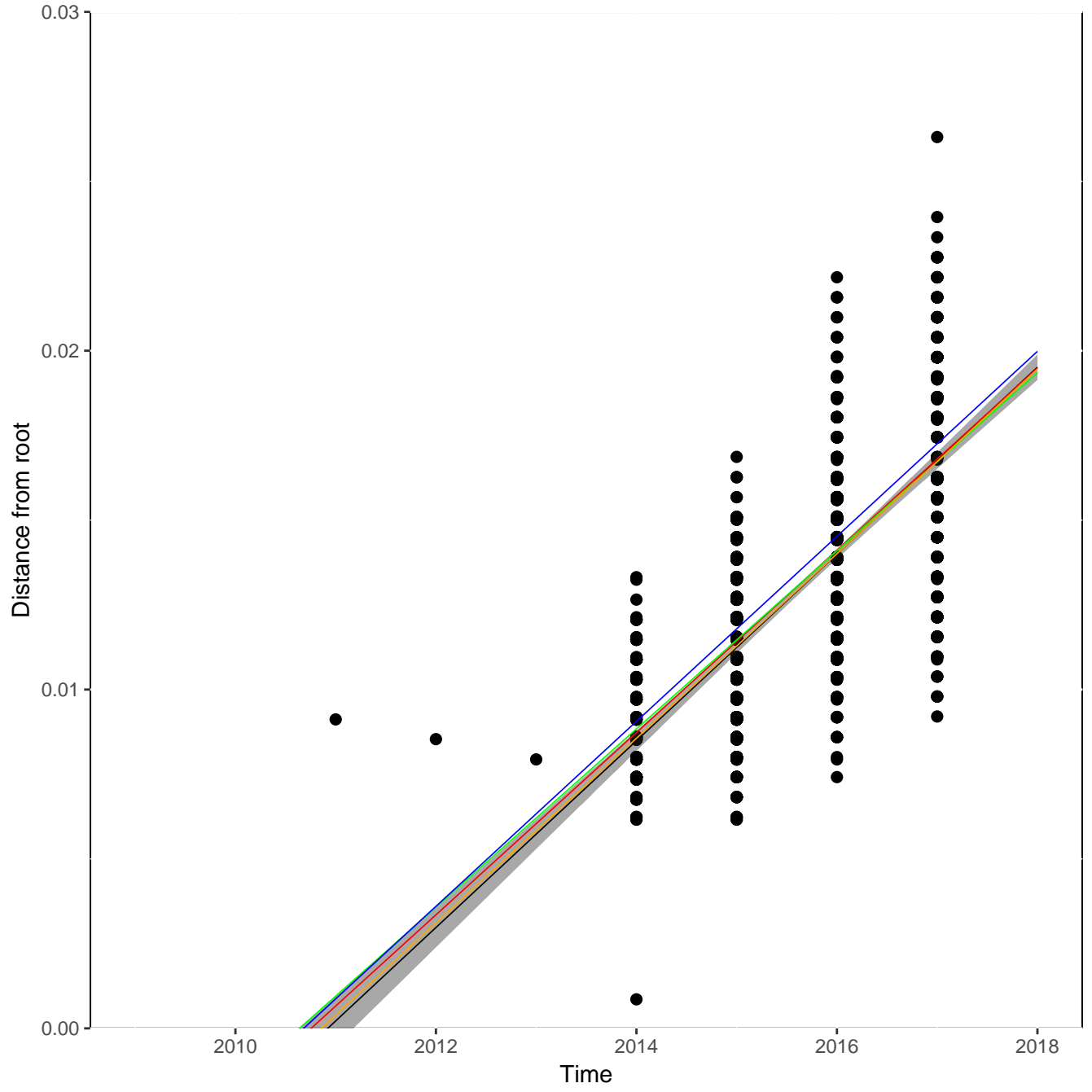

Supplement: Supplementary file 4 — Figure S1. Root-to-tip regression for the complete influenza dataset (2063 leaves, black dots and black regression line, in gray the 99% confidence interval) and four reduced trees (orange: 99% or RTL , red: 90% of RTL, green: 75% of RTL, blue: 50% of RTL. All trees were re-rooted with the best-fit method implemented in TempEst. (PDF 12 kb) [file 12859_2018_2164_MOESM4_ESM.pdf]

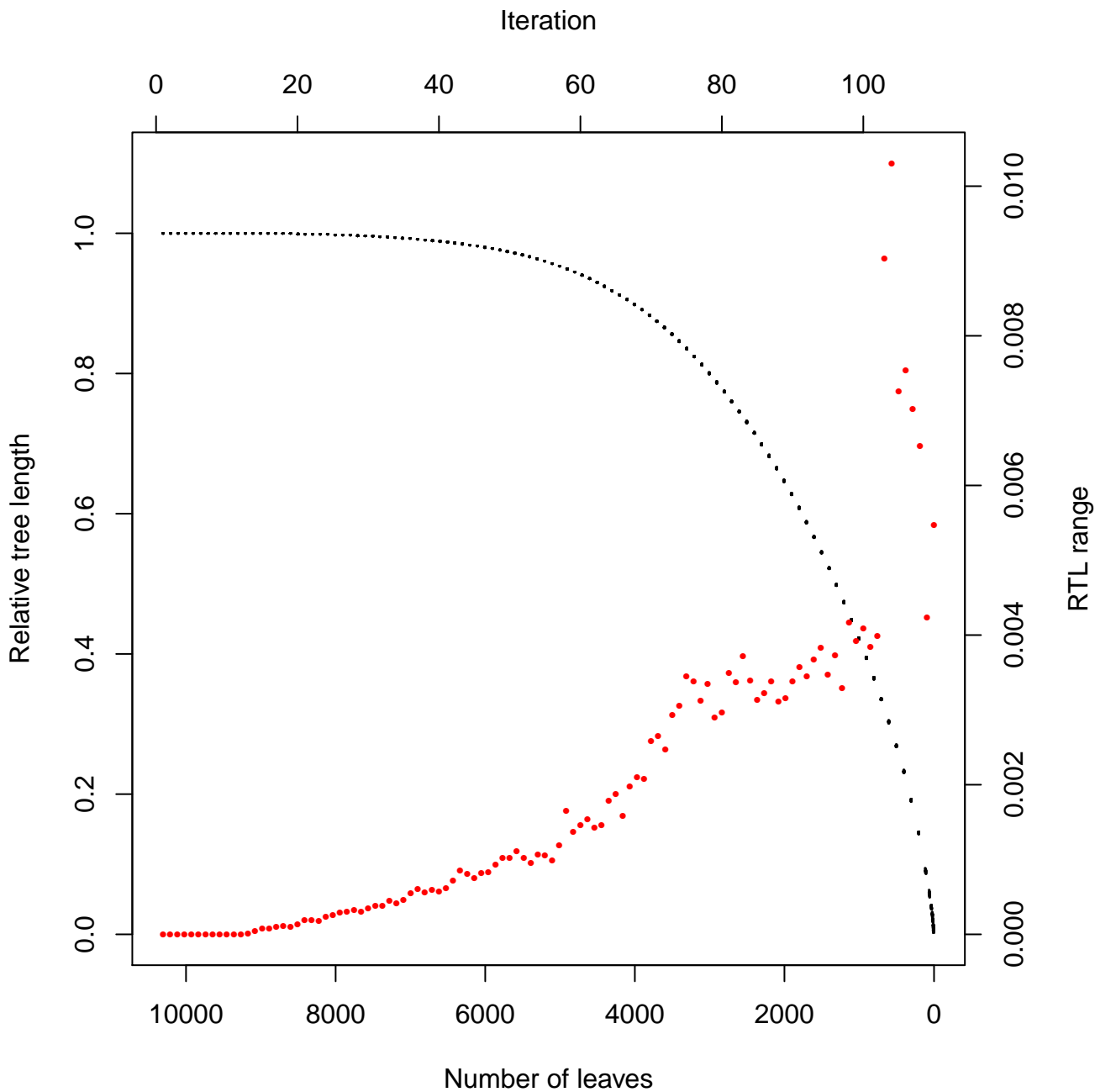

Supplement: Supplementary file 5 — Figure S2. Plot of 100 RTL decays (-r =100) for the TB dataset (black dots). All decays are similar. Red dots indicates the range of RTL among the different runs for the corresponding iteration, the variability among runs increases slowly at first, it reaches a maximum of 1% when the tree is reduced to 20% of the RTL. (PDF 25 kb) [file 12859_2018_2164_MOESM5_ESM.pdf]

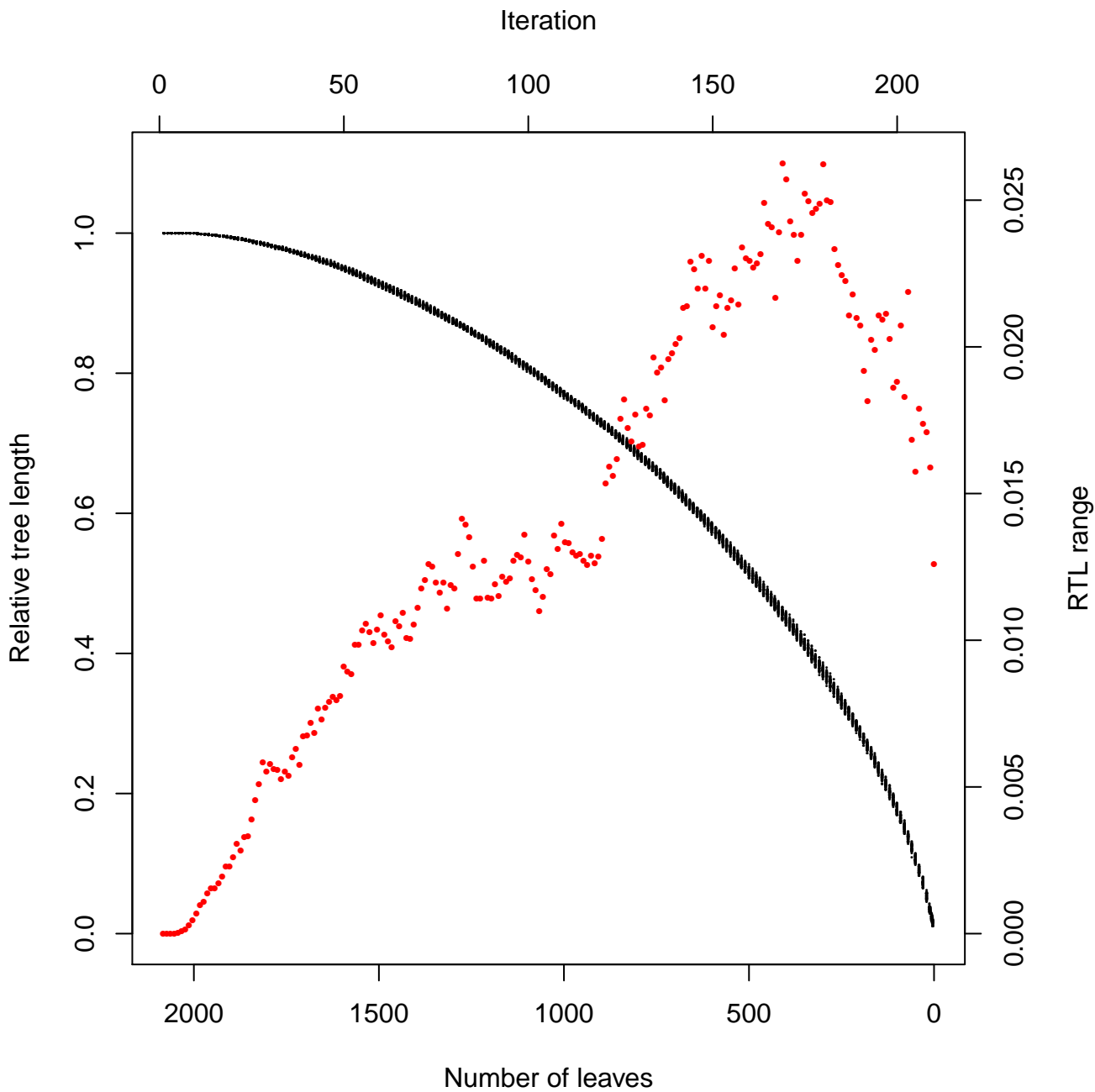

Supplement: Supplementary file 6 — Figure S3. Plot of 100 RTL decays (-r =10) for the Influenza dataset (black dots). All decays are similar, but there is a larger variability compared to the TB dataset. Red dots indicate the range of RTL among the different runs for the corresponding iteration, the variability among runs increases steadily and it reaches a maximum of 2.5% when the tree is reduced to 40% of the RTL. (PDF 56 kb) [file 12859_2018_2164_MOESM6_ESM.pdf]
